# Identification of calcium metabolism related score associated with the poor outcome in papillary thyroid carcinoma

**DOI:** 10.3389/fonc.2023.1108773

**Published:** 2023-03-28

**Authors:** Chuanxiang Hu, Lijuan Yan, Peng Li, Yang Yu

**Affiliations:** ^1^ Department of Thyroid and Neck Tumor, Tianjin Medical University Cancer Institute and Hospital, National Clinical Research Center for Cancer, Key Laboratory of Cancer Prevention and Therapy, Tianjin’s Clinical Research Center for Cancer, Tianjin, China; ^2^ State Key Laboratory of Medicinal Chemical Biology, Collaborative Innovation Center of Tianjin for Medical Epigenetics, Collaborative Innovation Center for Biotherapy, Nankai University, Tianjin, China; ^3^ Tianjin Key Laboratory of Protein Sciences, National Demonstration Center for Experimental Biology Education and College of Life Sciences, Nankai University, Tianjin, China

**Keywords:** calcium metabolism, genetic signature, functional analysis, poor outcome, papillary thyroid carcinoma

## Abstract

**Introduction:**

Papillary thyroid carcinoma is a type of thyroid cancer that exhibits significant variability in prognosis. Extensive research indicates that the impaired signaling of 1,25(OH)2D3-VDR may be a crucial factor in the development and progression of PTC.

**Methods:**

To investigate this further, Integrated analysis mRNA expression information from The Cancer Genome Atlas and GEO, we compared gene expression in cancer and normal tissues and identified differentially expressed genes (DEGs). Through this analysis, we identified DEGs and calculated risk estimates for seven genetic markers.

**Results:**

Subsequently, we constructed predictive models using LASSO-Cox regression to test the predictive value of these markers. Our results revealed that 64 calcium metabolism-related genes showed significant differences between tumor and normal tissues. Ten of the identified DEGs were significantly associated with overall survival, indicating their potential role in disease progression. Using the average risk score for the seven genetic markers, we divided patients into high- and low-risk groups. We found that patients in the low-risk group had significantly better overall survival than those in the high-risk group, highlighting the importance of these genetic markers in predicting prognosis. Further analysis using Cox regression demonstrated that the risk levels had independent predictive power. Additionally, we conducted functional analysis of the identified genetic markers, which showed significant differences in immune status between the two patient groups. We also investigated the effect of these calcium metabolism-related genes on thyroid cancer biological functions, immune microenvironment, and drug resistance.

**Discussion:**

Our findings provide evidence of a novel genetic signature associated with calcium metabolism, which can predict prognosis in patients with PTC. These results may have significant implications for the development of new diagnostic and therapeutic approaches to improve outcomes for PTC patients.

## Introduction

Among endocrine tumors, thyroid cancer is one of the most common (1). There has been a tripled increase in incidence over the past 25 years (2). The main thyroid cancer subtypes, including papillary thyroid carcinoma (PTC) which is the most common type of thyroid cancer accounting for about 80% of all cases, follicular thyroid carcinoma (FTC) which is less common than PTC accounting for about 10-15% of thyroid cancer cases, medullary thyroid carcinoma (MTC) which arises from the C cells of the thyroid gland, which produce a hormone called calcitonin and less common than PTC and FTC, accounting for about 5-10% of thyroid cancer cases, anaplastic thyroid carcinoma (ATC) which is the most aggressive type of thyroid cancer, accounting for less than 2% of cases. It typically arises from a pre-existing PTC or FTC, and can rapidly grow and spread to other parts of the body, thyroid lymphoma is a rare type of thyroid cancer that arises from lymphocytes, a type of white blood cell. Each of these thyroid cancer subtypes has distinct characteristics, treatment approaches, and outcomes (1, 3).

Papillary thyroid carcinoma (PTC) is the most common pathological form. Most PTC patients have a slow progression of disease and a good prognosis, with a 10-year survival rate>90% (4). However, it is worth noting that PTC is highly susceptible to lymph node metastasis. In a few cases, invasion and metastasis occurred at the early stage of the disease, and some even developed to advanced stage with systemic metastasis such as lung metastasis and bone metastasis, resulting in treatment failure and poor prognosis (5). It is estimated that more than 10% of PTC patients can eventually develop distant metastasis or cancer recurrence and may experience cancer-related death (6). The diagnosis of PTC is currently based on radiographic examination and post-operative pathology, and there is a lack of effective molecular markers for early detection and prognosis. As a result, we are unable to distinguish these invasive PTC from harmless PTC at an early stage. For the early diagnosis and the customization of individualized treatment plans of patients with PTC, the search for valuable predictive and prognostic indicators is now particularly important.

Intracellular calcium (Ca2+) is the most abundant messenger in the human body and plays a variety of roles in basic cell physiology, including gene expression, cell cycle regulation, cell motility, autophagy, and apoptosis (7). Prolonged elevated intracellular Ca2+ levels can lead to toxicity and induce cell death (8). Therefore, the Ca2+ signal must be tightly controlled in space and time (9).Intracellular Ca2+ oscillation is a powerful tool for transmitting biological information in cells. Previous studies have shown that intracellular Ca2+ fluctuations provide necessary growth signals for esophageal cancer cells (10). These intracellular Ca2+ oscillations affect gene transcription, cell proliferation, and migration in particular by their frequency, amplitude and duration (10, 11). Decoding of oscillatory patterns is performed by downstream intracellular effectors such as calmodulin (12), nuclear factor-activated T cells (NFAT), nuclear factor kappa b (NF-κb), calmodulin-dependent protein kinase II (CaMKII) and calpain. They activate and deactivate Ca2+ at different rates and then activate different cellular processes (13–15). Disruption of normal Ca2+ signaling results in a malignant phenotype. Tumors transform Ca2+ signaling networks in order to grow rapidly, increase cell survival and invasion, avoid death, avoid immune attack, or generate new blood vessels. It is increasingly recognized that oncogenic signaling pathways are associated with impaired expression levels or insufficient activation of Ca2+ channels, transporters, or Ca2+ ATPases (16–18). Altering these abnormal Ca2+ signals may have potential in cancer therapy.

In thyroid tumor cells, intracellular Ca2+ oscillations have been shown to play a role in the regulation of the target of rapamycin (mTOR) pathway, a key signaling pathway involved in cell growth and proliferation (19–21). Studies have shown that stimulation of thyroid tumor cells with thyrotropin-releasing hormone (TRH) induces Ca2+ oscillations, which in turn activate the mTOR pathway (22). In thyroid carcinoma, Wnt5a suppresses cell growth by phosphorylating β-catenin, acting as a tumor suppressor. Additionally, it has been shown that dysregulation of intracellular Ca2+ signaling can contribute to thyroid tumor development and progression. Mutations in the Ca2+ channels or pumps that regulate Ca2+ oscillations can disrupt normal signaling and contribute to tumor growth (23). Overall, the role of intracellular Ca2+ oscillations in thyroid tumor cell biology and the mTOR pathway is an active area of research, and further studies are needed to fully understand the mechanisms involved and to identify potential therapeutic targets for the treatment of thyroid cancer. In present study, we tried to figure out the potential relationship between calcium metabolism and PTC. The purpose of this study is to determine prognosis-associated tumor calcium metabolism related markers that predict the prognosis of PTC. We extracted the gene expression of PTC patients from the TCGA and GEO database, as well as their clinically relevant information. Through bioinformatics analysis, prognosis-associated calcium metabolism of PTC was screened, and the mechanisms of key calcium metabolism genes were further analyzed in order to construct a prognostic model for PTC. In conclusion, the results of this study indicate that Ca2+ metabolism-related genes are associated with the prognosis of PTC, and the seven genes identified in this study could potentially serve as biomarkers for predicting the prognosis of PTC. Valid predictors may provide individualized treatment and follow-up for patients with PTC and may come up with new ideas for novel therapeutic targets for PTC.

## Materials and methods

### Data collection

Quantitative gene expression and clinical features of PTC were obtained from The Cancer Genome Atlas (TCGA, http://portal.gcd.cancer.gov/) and the Gene Expression Comprehensive Database Portal (GEO, https://www.ncbi.nlm.nih.gov/geo/query/acc.cgi). Gene expression data were normalized using the ‘limma’ R package. This study has not been ethically reviewed and is in compliance with TCGA ethics and guidelines, as all data used are public.

### Searching for differentially expression genes

According to the GSEA database, calcium metabolism family genes were selected for further analysis (**Table 1**). Wilcoxon test was used to identify differentially expressed genes in PTC and R normal tissues. We searched for differentially expressed genes related to calcium metabolism from all DEG tumors and cis-division data. The DEG distribution between PTC and normal thyroid tissues was depicted in a volcano plot. Changes in expression of DEGs, calcium metabolism related between PTC and thyroid normal tissues are represented by heat maps.

**Table 1:** Calcium metabolism family genes from GSEA database.

Calcium metabolism family genes
ADCY1 ADCY2 ADCY3 ADCY4 ADCY7 ADCY8 ADCY9 ADORA2A ADORA2B ADRA1A ADRA1B ADRA1D ADRB1 ADRB2 ADRB3 ATP2A2 ATP2A3 ATP2B1 ATP2B2 ATP2B3 ATP2B4 AVPR1A AVPR1B BDKRB1 BDKRB2 BST1 ATP2A1 CACNA1A CACNA1B CACNA1C CACNA1D CACNA1E CACNA1F CACNA1G CACNA1H CACNA1I CACNA1S AGTR1 CALM1 CALM2 CALM3 CALML3 CALML5 CALML6 CAMK2A CAMK2B CAMK2D CAMK2G CAMK4 CCKAR CCKBR CD38 CHP1 CHP2 CHRM1 CHRM2 CHRM3 CHRM5 CHRNA7 CYSLTR1 CYSLTR2 DRD1 DRD5 EDNRA EDNRB EGFR ERBB2 ERBB3 ERBB4 F2R GNA11 GNA14 GNA15 GNAL GNAQ GNAS GRIN1 GRIN2A GRIN2C GRIN2D GRM1 GRM5 GRPR HRH1 HRH2 HTR2A HTR2B HTR2C HTR4 HTR5A HTR6 HTR7 ITPKA ITPKB ITPR1 ITPR2 ITPR3 LHCGR LTB4R2 MYLK MYLK2 MYLK3 NOS1 NOS2 NOS3 NTSR1 OXTR P2RX1 P2RX2 P2RX3 P2RX4 P2RX5 P2RX6 P2RX7 PDE1A PDE1B PDE1C PDGFRA PDGFRB PHKA1 PHKA2 PHKB PHKG1 PHKG2 PLCB1 PLCB2 PLCB3 PLCB4 PLCD1 PLCD3 PLCD4 PLCE1 PLCG1 PLCG2 PLCZ1 PLN PPID PPP3CA PPP3CB PPP3CC PPP3R1 PPP3R2 PRKACA PRKACB PRKACG PRKCA PRKCB PRKCG PRKX PTAFR PTGER1 PTGER3 PTGFR PTK2B RYR1 RYR2 RYR3 SLC25A31 SLC25A4 SLC25A5 SLC25A6 SLC8A1 SLC8A2 SLC8A3 SPHK1 SPHK2 TACR1 TACR2 TACR3 TBXA2R TNNC1 TNNC2 TRHR TRPC1 VDAC1 VDAC2 VDAC2P5 VDAC3

### Searching for prognostic calcium metabolism related DEGs

To investigate the prognostic value of calcium-related DEGs in patients with PTC, Univariate Cox regression were performed. Inclusion criteria for patients is that whose observation period is longer than 90 days to ensure the effect stronger. Gene with P<0.005 were marked significant and shown in forest plots. Furthermore, a hazard ratio >1 indicates a high-risk gene and an HR >1 indicates a low-risk gene.

### Construction and validation of genetic signatures associated with calcium metabolism

Cox analysis helps to study the ability of calcium-related genes to predict overall survival (1). LASSO-Cox regression is a powerful variable selection and fitting method, performed using the “limma” R package, to analyze whether DEG can predict OS and status in PTC patients. The penalty parameter λ is determined by 10-fold cross-validation according to the optimal value of the minimum likelihood ratio.

Risk scores were calculated according to the following formula: Risk Score = esum (normalized expression of each gene × its regression coefficient). The mean risk score was used as a criterion for classifying patients into high- and low-risk groups. We used PCA and t-SNE and the R packages to describe the distribution of the two groups of genes. For survival analysis, the cutoff value for gene expression was obtained with the “surv_cutpoint” function of the R package. Then, we performed a time-dependent ROC analysis using the “survivalROC” R package to assess whether this gene signature had predictive power. Univariate and multivariate Cox regression analyses were then used to determine whether the risk score could independently predict patients’ OS prognosis.

### Functional enrichment analysis

Gene Ontology (GO) enrichment and Kyoto Encyclopedia of Gene and Genomes (KEGG) pathway analysis were performed on the two sets of DEGs using the R package with |log2 (fold change) | ≥ 1 and FDR < 0.05. Meets DEG assessment criteria. The “gsva” R package was used for Single Gene Set Enrichment Analysis (ssGSEA) to calculate enrichment scores for immune cells and immune-related signaling pathways.

### Evaluation of tumor immune microenvironment

Generation of immune score and StromaScore and calculation of cell type immune score: Using the ESTIMATE software package, the ratio of immune and framework components in the tumor microenvironment for each sample was determined and reported as two values, StromaScore and immune score. The higher the score, the greater the percentage within the tumor micro- environment.

Ratios of 22 immune cell types (neutrophils, eosinophils, activated mast cells, resting mast cells, activated dendritic cells, resting dendritic cells, macrophages M2, macrophages M1, macrophages M0, monocytes, activated NK cells, resting NK cells, and so on) between high- and low-risk patients were assessed using the CIBERSORT method.

### Nomogram model establishment

**Figure 1:**
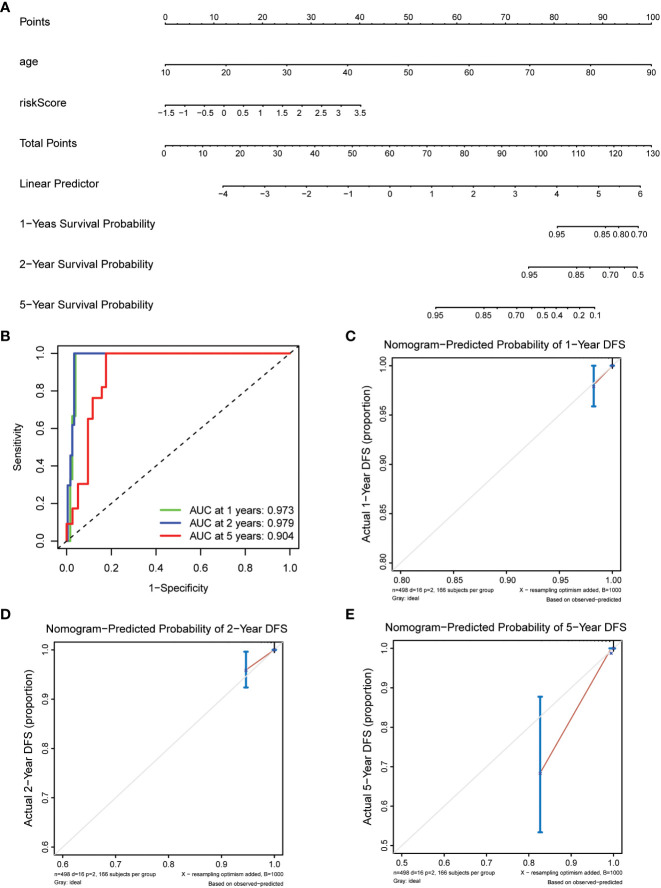
Construction of nomogram models for thyroid cancer. **(A)** A nomogram combining the risk score and age. **(B)** AUC of time-dependent ROC curves evaluated the prognostic capacity of the nomogram. **(C–E)** Calibration curves comparing the nomogram-predicted **(C)** 1-, **(D) **2-, and **(E)** 5-year survival and actual survival.

Prepare nomogram infographics by combining age and risk analysis. Calculate the total score for each patient according to **Figure 1A**. The predicted survival rates from the 1-, 2-, and 5-year survival nomograms were compared to the actual survival rates from the standard curve.

### Drug-signature gene interaction

We looked for drugs that respond to promising target genes marked by multiple genes. This study was conducted using the Drug-Gene Interaction Database (https://discover.nci.nih.gov/cellminer/loadDownload.do).

## Results

In this study, we created a prognostic model using calcium metabolism-related DEGs to investigate the effect of calcium metabolism-related genes on PTC prognosis, and validated the model with extrinsic and tissue validation. Moreover, we also investigate the affect on thyroid cancer biological functions, immune microenvironment and drug resistance of these calcium metabolism-related genes.

### Identification of calcium metabolism-related DEGs with predictive value

**Figure 2:**
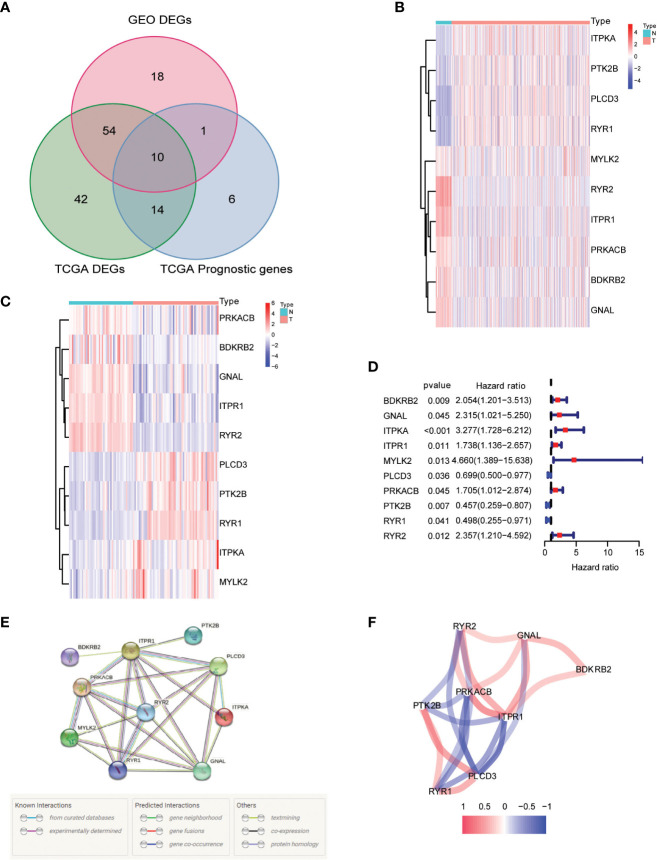
Identification of calcium metabolism related differentially expressed genes. **(A)** Venn plot of the differentially expressed genes between tumor and normal tissue that were correlated with OS. **(B, C)** Heatmaps of the differentially expressed genes associated with OS. **(D)** Forest plot of the results of the univariate Cox regression analysis between gene expression and OS. **(E, F)** The correlation of the differentially expressed genes associated with OS.

Our research began with a comprehensive analysis of two large-scale databases, TCGA and GEO (GSE33630), which revealed a wealth of valuable data on the differential expression of calcium metabolism-related genes in tumors and adjacent normal tissues (**Figure 2A**). Intriguingly, our results showed that 64 genes exhibited distinct patterns of expression between the two groups, providing compelling evidence for the involvement of these genes in the development and progression of papillary thyroid carcinoma (PTC). Further exploration of the TCGA database revealed that 10 of these genes were specifically related to patient overall survival (OS), underscoring their potential as valuable prognostic markers (**Figure 2A**). In-depth examination of the differential gene expression profiles was facilitated by heatmaps generated from both TCGA and GEO datasets, which vividly highlighted the marked differences between tumor and normal tissue (**Figures 2B, C**). Furthermore, we constructed a forest plot to demonstrate the relationship between the expression of these calcium metabolism-related genes and patient survival outcomes (**Figure 2D**). Notably, we also observed strong correlations between the expression levels of these genes, as depicted in our striking visual representation of the interrelationships between them (**Figures 2E, F**). Taken together, our findings provide a comprehensive framework for understanding the complex roles of calcium metabolism-related genes in the pathogenesis of PTC and open new avenues for exploring their potential as targets for diagnosis and treatment.

### Prognostic model construction

**Figure 3:**
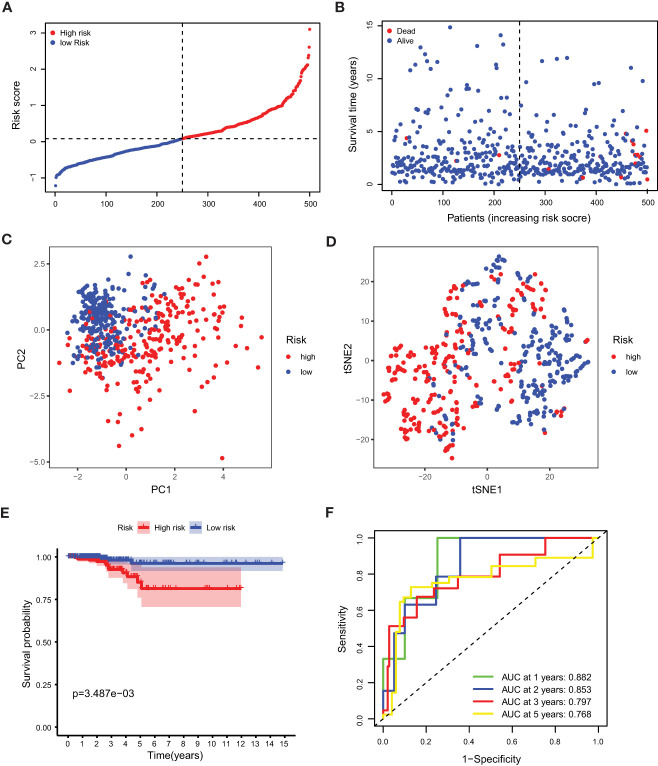
Prognostic analysis of the 5-gene signature model. **(A)** The distribution and median value of the risk scores. **(B)** The distributions of OS status, OS and risk score. **(C)** PCA analysis of the TCGA cohort. **(D)** t-SNE analysis of the TCGA cohort. **(E)** Kaplan-Meier curves of the OS in the two groups. **(F)** AUC of time-dependent ROC curves evaluated the prognostic capacity of the risk score.

**Figure 4:**
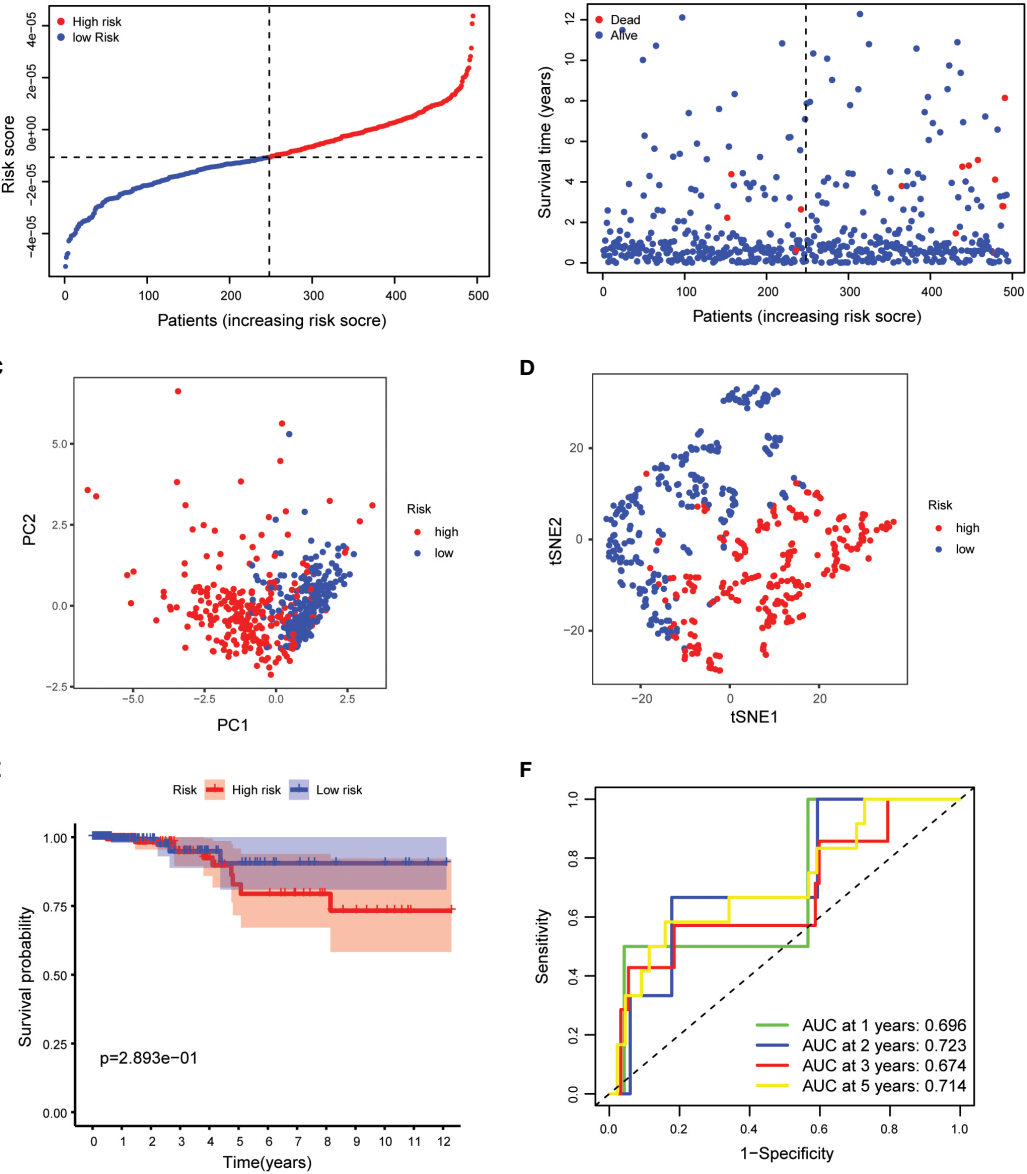
Validation of the 5-gene signature model. **(A)** The distribution and median value of the risk scores. **(B)** The distributions of OS status, OS and risk scores. **(C)** PCA analysis of the ICGC cohort. **(D)** t-SNE analysis of the ICGC cohort. **(E)** Kaplan-Meier curves of the OS in the two groups. **(F)** AUC of time-dependent ROC curves evaluated the prognostic capacity of the risk score.

Our study took a crucial step forward by developing a sophisticated prognostic model using 10 calcium metabolism-related genes that were significantly associated with overall survival (OS) in papillary thyroid carcinoma (PTC) patients. To achieve this, we applied LASSO-Cox regression analysis to generate a risk score for each patient, which was based on the expression levels of seven genes (BDKRB2, ITPKA, ITPR1, MYLK2, PTK2B, RYR1, and RYR2). The formula used for calculating the risk score was as follows: Risk score= (0.339 * BDKRB2 + 1.081 * ITPKA + 0.144 * ITPR1 + 0.701 * MYLK2 - 0.342 * PTK2B - 0.063 *RYR1 + 0.109 *RYR2). We then classified patients into low- and high-risk groups based on the mean risk score. Remarkably, we found that patients in the high-risk group had significantly higher mortality rates than those in the low-risk group, as demonstrated by our compelling data presented in **Figures 3A, B**. To further validate our findings, we performed principal component analysis (PCA) and t-distributed stochastic neighbor embedding (t-SNE) analysis, which revealed a bidirectional distribution of PTC patients in the two groups (**Figures 3C, D**). Importantly, the Kaplan-Meier curve confirmed that the OS of the low-risk group was significantly higher than that of the high-risk group (**Figure 3E**, p< 0.05). The predictive power of the OS risk scores was demonstrated by the areas under the curve of 0.882, 0.853, 0.797, and 0.768 at 1, 2, 3, and 5 years, respectively (**Figure 3F**). Furthermore, to confirm the reliability of our 7-gene signature, we performed additional validation using another database, ICGC, and obtained similar results (**Figures 4A–F**). In summary, our novel prognostic model using calcium metabolism-related genes provides a valuable tool for predicting patient survival and may guide personalized treatment decisions for PTC.3.3 Risk score for independent predictive power.

**Figure 5:**
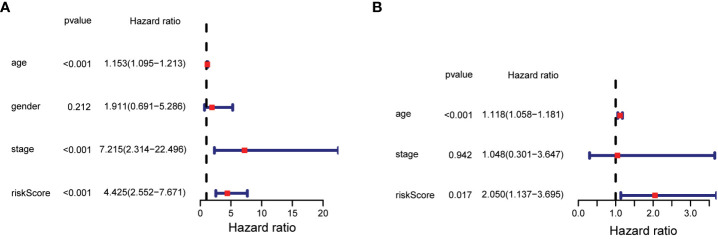
Results of univariate and multivariate Cox regression analysis on OS. **(A)** Univariate Cox regression analysis on OS. **(B)** Multivariate Cox regression analysis on OS.

The statistical significance of the prognostic model was further evaluated by Cox regression analysis. Univariate Cox regression analysis demonstrated that the risk score based on the 7-gene signature was strongly associated with OS (hazard ratio (HR) = 4.425, 95% confidence interval (CI) = 2.552-7.671, p < 0.001) (**Figure 5A**). This result was supported by the multivariate Cox regression analysis, which showed that the risk score was an independent predictor of OS (HR = 2.050, 95% CI = 1.137-3.695, P = 0.017) (**Figure 5B**). The results indicated that the prognostic model based on the 7-gene signature could be used as an independent and reliable prognostic tool for PTC patients.3.4 Development of a nomogram model for thyroid cancer.

To enhance the understanding of patient outcomes, we developed a survival nomogram model that integrated two independent prognostic factors, namely age and risk score, to predict 1-, 2-, and 5-year survival rates in patients with thyroid cancer (**Figure 1A**). The nomogram demonstrated excellent predictive accuracy, with areas under the curve of 0.973, 0.979, and 0.904 at 1, 2, and 5 years, respectively, indicating strong discrimination ability (**Figure 1B**). Furthermore, the calibration curves showed that the predicted survival probabilities were closely aligned with actual survival rates, demonstrating high consistency and reliability (**Figures 1C–E**). Overall, our nomogram model provides a powerful and personalized tool for accurately predicting survival probabilities in patients with thyroid cancer, which could help guide clinical decision-making and optimize patient management.

### Functional analysis in TCGA

**Figure 6:**
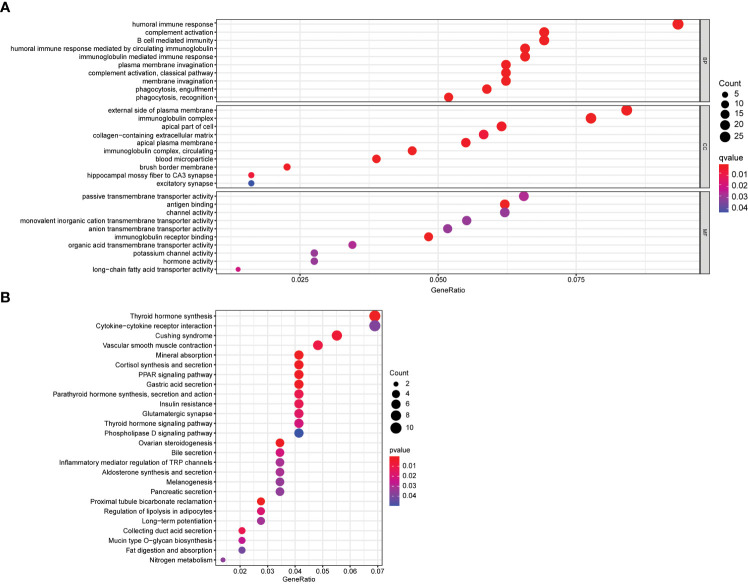
Functional enrichment analysis of DEGs. **(A)** Top 10 biological process (BP) terms, cellular components terms (CC), molecular functions (MF) terms. **(B)** Top 26 Kyoto Encyclopedia of Genes and Genomes (KEGG) pathways.

To gain insight into the biological functions and pathways involved in risk assessment, we performed a comprehensive analysis of the differentially expressed genes (DEGs) using graphene oxide enrichment and KEGG pathway analysis. Our results revealed membrane deepening, circulating immunoglobulin-mediated humoral immune responses, immunoglobulin complexes, immunoglobulin receptor binding, and antigen binding as the top 10 BP, CC, and MF terms enriched by graphene oxide (**Figure 6A**). Additionally, KEGG pathway analysis identified 26 key pathways, including thyroid hormone synthesis, cytokine-cytokine receptor interaction, Cushing syndrome, vascular smooth muscle contraction, mineral absorption, and cortisol synthesis and secretion, among others (**Figure 6B**). These findings provide important insights into the mechanisms and pathways involved in thyroid cancer progression, and may contribute to the development of more effective therapeutic strategies for this disease.3.6 Association between calcium metabolism related model and immune cells

**Figure 7:**
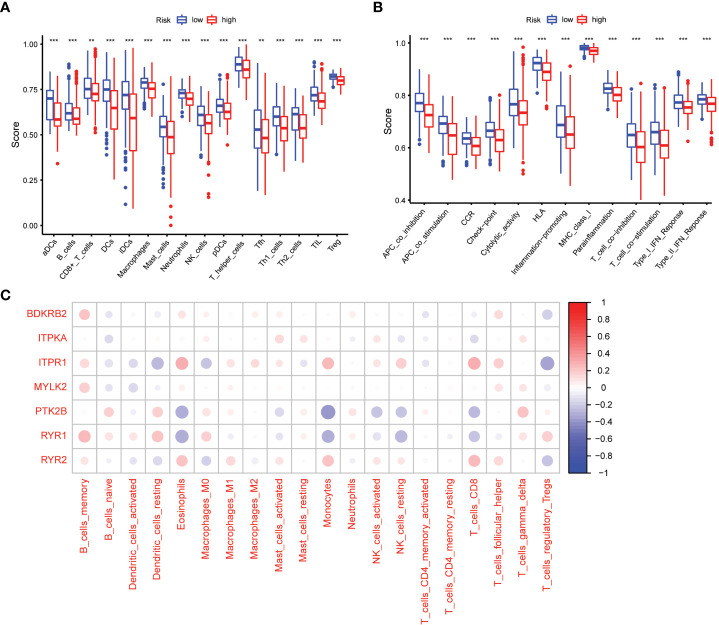
Comparison of the ssGSEA scores between different risk groups. **(A)** The scores of 16 immune cells. **(B)** The scores of 13 immune-related functions. Adjusted P values were showed as: **, P< 0.01; ***, P< 0.001. **(C)** The association between 7 prognostic calcium metabolism related genes and immune cells.

We used the ssGSEA cumulative score to quantify immune status and analyze its relationship to risk scores. The differences in accumulation rate of some immune cells and immune status between the two groups were statistically significant (**Figures 7A, B**).

To explore the relationship between the immune microenvironment and calcium metabolism-related genes included in the prognostic model, we conducted a correlation analysis. Our results revealed that BDKRB2, ITPKA, ITPR1, MYLK2, PTK2B, RYR1, and RYR2 were associated with the function of immune cells. Notably, immune cells such as memory B cells, eosinophils, macrophages M1, monocytes, activated NK cells, CD8+ T cells, and follicular helper T cells exhibited positive correlations with ITPR1 and RYR2. These findings suggest that the 7 calcium metabolism-related genes in our prognostic model are involved in regulating the immune microenvironment (**Figure 7C**).

### Gene expression interacted with drug treatment

**Figure 8:**
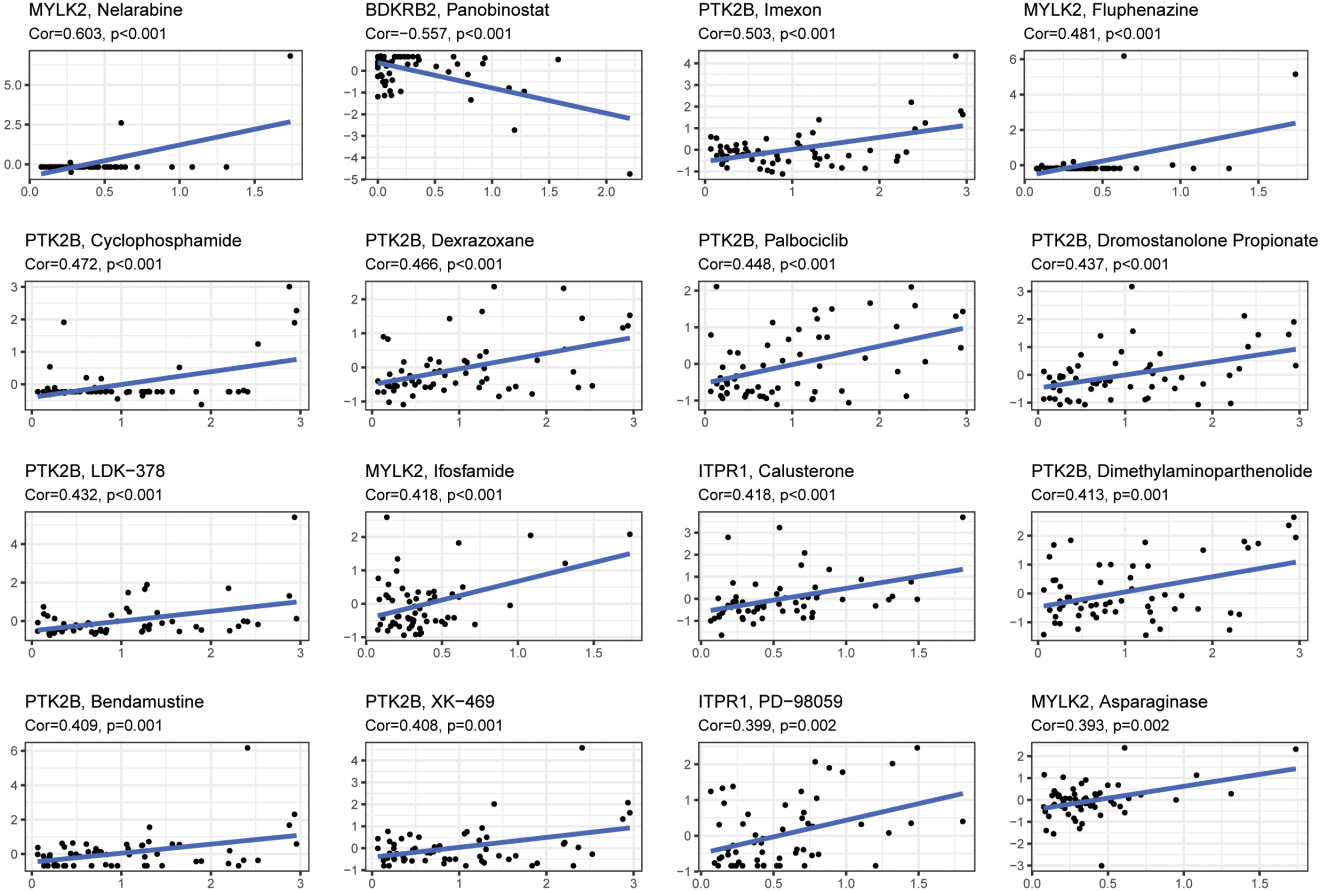
The correlation between gene expression levels and drugs. The top 16 most relevant were visualized.

The relationship between level of 4 signature genes (MYLK2, BDKRB2, PTK2B and ITPR1) and 16 drugs were explored (**Figure 8**). Among these genes, PTK2B was up regulated in response to drugs treatment significantly. MYLK2 was up regulated by Nelarbine, Fluphenazine, Ifosfamide and Asparaginase treatment. ITPR1 showed significant correlation with Calusterone and PD-98059. BDKRB2 showed down regulation upon panobinostat treatment. The drug data was obtained from the cellminer database (https://discover.nci.nih.gov/cellminer/loadDownload.do).

## Discussion

In the past, molecular mechanisms regulating intracellular calcium (Ca2+) influx has received limited attention as a potential target for cancer therapy, and therefore not considered to be relevant to developing selective formulations (24). However, over the past decade, the roles of Ca2+ in tumor transformation, tumor progression, and treatment response were reassessed (25). Thus, the molecular mechanisms controlling Ca2+ signaling in tumor cells were found to be altered due to their importance of changes in expression levels and post-translational modifications or interacting components (7). These defects can lead to malignant changes, promote tumor progression and play important therapeutic roles. Therefore, we tried exploring the correlation between calcium metabolism related genes and PTC in this study.

Calcium (Ca2+) signaling is a critical process in many biological functions, including muscle contraction, neurotransmitter release, and gene expression (8, 26, 27). Ca2+ signaling is mediated by a variety of transduction channels that allow the passage of Ca2+ ions across the cell membrane or intracellular membranes. These channels are classified based on their location and function, and include voltage-gated channels, ligand-gated channels, store-operated channels, and second messenger-operated channels (28). Voltage-gated channels are activated by changes in membrane potential, and are important for the generation and propagation of action potentials in excitable cells. Ligand-gated channels are activated by the binding of a specific ligand, such as a neurotransmitter or hormone (29, 30). Store-operated channels are activated by depletion of intracellular Ca2+ stores, and play a critical role in Ca2+ homeostasis (31). Second messenger-operated channels are activated by second messengers, such as inositol triphosphate (IP3) or cyclic ADP-ribose (cADPR), and are important for regulating Ca2+ release from intracellular stores. Dysregulation of Ca2+ signaling transduction channels can lead to a variety of diseases, including cardiac arrhythmias, muscular dystrophies, and neurodegenerative disorders (19, 32–34). Therefore, understanding the structure and function of these channels is crucial for developing new therapies to treat these conditions.

Calcium plays a critical role in several cellular processes, including cell proliferation, differentiation, and apoptosis. In thyroid cells, calcium signaling pathways are known to be involved in regulating thyroid hormone synthesis and secretion (35). Dysregulation of calcium homeostasis has been linked to the development and progression of several types of cancer, including PTC (36). Studies have shown that alterations in calcium metabolism, such as changes in the expression or activity of calcium channels, pumps, and exchangers, can lead to the development of PTC (12, 37, 38). For example, mutations in genes encoding calcium channels and pumps, such as TRPC3, TRPM7, and SERCA2, have been identified in PTC patients (39, 40). These mutations have been associated with an increased risk of tumor recurrence and a poorer prognosis. Furthermore, recent research has suggested that the genetic signature of altered calcium metabolism may be associated with a poorer prognosis in PTC patients (12). This suggests that calcium metabolism may play a central role in the development, progression, and prognosis of PTC. Understanding the role of calcium metabolism in PTC is important for developing new therapies that target these pathways. For example, drugs that modulate calcium channels or pumps may be effective in treating PTC. Additionally, identifying genetic markers of altered calcium metabolism could help predict the prognosis of PTC patients and guide treatment decisions. Overall, further research into the role of calcium metabolism in PTC could provide new insights into the molecular mechanisms of this disease and improve patient outcomes.

Based on the GEO and TCGA databases, differential gene expression analysis showed that 10 calcium metabolism-related genes were differentially expressed in tumors and adjacent normal tissues, and these genes were significantly associated with OS. Prognostic models were constructed by LASSO Cox regression analysis, and then a 7-gene signature which were associated with OS was determined according to the optimal value of λ. Risk analysis, survival analysis, ROC curve analysis and independent prognostic analysis were used to verify the feasibility of the model. Through a series of analyses, our study confirms that calcium metabolism related DEGs are closely associated with the prognosis of PTC. The construction of a prognostic model of PTC with 7 genes provides a new diagnostic and therapeutic target for potential therapy of PTC. The results showed that these 7 genes may be feasible predictors of PTC prognosis, including BDKRB2, ITPKA, ITPR1, MYLK2, PTK2B, RYR1 and RYR2. These seven genes are interconnected in various ways.

BDKRB2 is a 9-amino acid bradykinin receptor that is constitutively expressed in blood vessels. Bradykinin is a positive regulator of angiogenesis. BDKRB2 is overexpressed in many human cancers (41). BDKRB2 promotes tumor growth and invasion, increases blood supply and other survival signals (28). Furthermore, it interacts with α5β1 integrin to alter the transcriptional activation of epidermal growth factor receptor in renal cells (42). BDKRB2 mediates the induction of cyclooxygenase-2 and endothelin-1, which act as stimulators of angiogenesis (43).

In recent years, increasing evidence has shown that ITPKA is overexpressed and acts as a promoter in a variety of cancers, including breast, lung, and liver cancer (44–46). ITPKA is reported to be a bifunctional protein that phosphorylates Ins (1,4,5) P3 *via* kinase activity and cross-links f-actin *via* f-actin-binding activity, which accounts for the ITPKA-promoting effects on cancer development (47). ITPKA can promote the growth, migration and invasion of renal cell carcinoma (RCC) by activating the m-TORC1 signaling pathway. This provides a new mechanism by which ITPKA regulates RCC growth and development (48). In PTC, ITPR1 expression has been found to be upregulated, suggesting that it may contribute to the dysregulation of calcium signaling pathways in this cancer (49).

MYLK2 encodes for myosin light chain kinase 2, an enzyme that is involved in the regulation of muscle contraction and cell adhesion. MYLK3 is a protein-coding gene on chromosome 16 (16q11.2) known for its role in regulating the actin cytoskeleton and signaling immune responses. It phosphorylates the heavy chain (MYH7B) and light chain (MYL2) of cardiac myosin, thereby increasing the strength and recruitment rate of crosslinks in cardiomyocytes. Myosin light chain kinase is involved in the regulation of epithelial cell survival, and knockdown of this gene leads to apoptosis of mammary epithelial cells *in vitro*. Chromosome 3 myosin light chain kinase is involved in epithelial tight junction permeability and has been shown to be an early warning marker for gastric cancer and promote cell proliferation (50–52).

PTK2B encodes for protein tyrosine kinase 2 beta, a signaling protein that is involved in the regulation of cell proliferation, migration, and survival. In PTC, PTK2B expression has been found to be upregulated, suggesting that it may contribute to the growth and survival of cancer cells. PTK2B was validated using clinical biopsies showing significant overexpression in aggressive compared to non-aggressive PTC (53).

Ryanodine receptors (RyR) are cation channels with high conductivity that release Ca2+ from intracellular stores such as the endoplasmic reticulum and sarcoplasmic reticulum (54). RyR1 is most expressed in the terminal SR pool of skeletal muscle (55). In fact, it has been well characterized as downstream of the STAT3 signaling pathway, which leads to the accumulation of breast cancer stem cells and increases the likelihood of tumor recurrence or metastasis (38). RyR2 is the first and most expressed biochemically isolated cardiac SR in mammals (56). Due to the biphasic effects of intracellular Ca2+ on cell survival and death, changes in RyR expression or function are important for tumor progression and/or therapy. For example, a paclitaxel-resistant subclone of human lung adenocarcinoma A549 has reduced RyR expression and function compared to the parental cell line (57). Likewise, RyR expression levels are positively correlated with breast cancer tumor stage (58). In contrast, *in vitro* experiments showed that the RyR1 antagonists dantrolene and azumolene induce cell death in low-grade B non-Hodgkin’s lymphoma (59). This may contribute to the dysregulation of calcium signaling pathways in this cancer.

Overall, these genes are interconnected in various ways through their involvement in calcium signaling pathways and cellular processes such as cell proliferation, migration, and survival. Dysregulation of these pathways in PTC may contribute to the development and progression of this cancer. Due to the novelty of the model, the accuracy of the model needs to be subsequently validated in clinical practice, and large sample data are needed to analyze the correlation between the model and TNM staging of PTC patients to validate the feasibility of the model.

The relationship between papillary carcinoma (the most common type of thyroid cancer) and T and B cell subtypes in the thyroid parenchyma is still an area of active research, and there is not yet a clear consensus on the predictive relationship between these factors. Some studies have suggested that the presence of intertumoral T cells may be associated with a better prognosis for papillary thyroid carcinoma, while others have reported no significant association between T cell infiltration and prognosis. Additionally, some studies have reported an association between the presence of B cells in the thyroid parenchyma and a more aggressive form of papillary thyroid carcinoma, while others have not found a significant association. It is important to note that the relationship between the immune system and cancer is complex and multifaceted, and many factors can influence the interaction between tumor cells and immune cells. For example, the presence of certain immune cell subtypes may be influenced by the genetic profile of the tumor, the tumor microenvironment, and other host factors. Overall, while there is some evidence to suggest that the presence of T and B cell subtypes in the thyroid parenchyma may be predictive of prognosis for papillary thyroid carcinoma, more research is needed to fully understand the complex interplay between the immune system and thyroid cancer, and to identify potential therapeutic targets for the treatment of this disease.

Recent studies have highlighted the importance of the tumor immune microenvironment (TIME) in cancer development and progression (60–62). Calcium metabolism genes have been shown to play a critical role in regulating the TIME. These genes encode proteins involved in calcium signaling pathways, such as the calcium-sensing receptor (CaSR), calcium channels, and calcium-binding proteins (31, 63–65). Dysregulation of these genes can lead to alterations in calcium homeostasis, which can affect immune cell function and, in turn, impact tumor growth and metastasis. The expression of sarcoplasmic reticulum Ca2+/ATPases (SERCA) can play a critical factor for cell/tumor homeostasis. SERCA pumps are responsible for regulating intracellular calcium levels, which are important for a variety of cellular functions such as muscle contraction, signaling pathways, and gene expression (66–68). In tumor cells, changes in SERCA expression and activity can lead to altered calcium signaling, which has been implicated in various aspects of cancer development and progression, including cell proliferation, apoptosis, migration, and invasion (69, 70). For example, decreased SERCA expression or activity has been associated with increased invasiveness and metastasis in several types of cancer (71, 72). Conversely, increasing SERCA expression or activity can enhance cellular stress responses, including the unfolded protein response and autophagy, which can help maintain cell homeostasis and suppress tumor growth (73, 74). Therefore, SERCA expression and activity are important for maintaining proper cellular function and may play a critical role in tumor cell homeostasis. Moreover, alterations in calcium channels and calcium-binding proteins have also been linked to changes in immune cell function and tumor development (28). In conclusion, the findings of recent studies suggest that calcium metabolism genes play an essential role in regulating the TIME and impacting tumor growth and metastasis. Further research is needed to fully understand the underlying mechanisms and develop new strategies to target these genes for cancer treatment.

Since the mechanism of calcium metabolism in tumors is not clear, we performed GO enrichment and KEGG pathway analysis. We found plasma membrane invagination, humoral immune response mediated by circulating immunoglobulin, thyroid hormone synthesis and cytokine−cytokine receptor interaction were enriched, which points a new direction for future research. However, our current results are limited by the use of public databases. Further studies are needed to validate the functional and mechanisms of the calcium metabolism related genes in thyroid cancer development. As to the four signature genes, MYLK2, BDKRB2, PTK2B, and ITPR1, are involved in various cellular processes and signaling pathways, and their expression levels have been implicated in several diseases. These genes may be used as biomarkers for various therapeutic interventions. The related 16 drugs mentioned in results are known to target different cellular pathways and may affect the expression or activity of these genes to varying degrees. PTK2B was found to be upregulated in response to drug treatments, indicating its potential as a therapeutic target for certain diseases (75). Additionally, some drugs may have different effects on the expression of these genes depending on the specific cell type or disease state. Therefore, the relationship between the expression levels of these genes and the effects of these drugs would need to be studied in the specific context of the disease and drug being considered. Our results provide a predictive model to show the potential relationship between biomarkers and therapeutic interventions.

## Data availability statement

The datasets presented in this study can be found in online repositories. The names of the repository/repositories and accession number(s) can be found in the article/supplementary material.

## Ethics statement

The studies involving human participants were reviewed and approved by Tianjin Medical University Cancer Institute and Hospital. Written informed consent for participation was not required for this study in accordance with the national legislation and the institutional requirements. Written informed consent was not obtained from the individual(s) for the publication of any potentially identifiable images or data included in this article.

## Author contributions

CH and LY download data, analyzed data, and contributed to the paper writing. PL and YY design the project and wrote the paper. All authors contributed to the article and approved the submitted version.
